# The Dialectics of Disembedding and Civil Society in Provincial Hungary

**DOI:** 10.1080/09668136.2021.1991891

**Published:** 2021-11-12

**Authors:** Chris Hann

## Abstract

This essay applies Karl Polanyi’s concepts of embedding and countermovement to provincial Hungary during and after socialism. Comprehensive state socialist repression in the 1950s was a politics-led disembedding. An economy-led countermovement began in the 1960s, later augmented by elite discourses of civil society. The 1970s and 1980s were decades of socialist embeddedness. Neoliberal configurations after 1990 dislocated both economic and associational life. The illiberal democracy of Viktor Orbán is a more consequential countermovement than the earlier countermovements to state socialism. The argument is illustrated with data from long-term fieldwork in southern Hungary, in the region of the Danube-Tisza Interfluve.

Scholars of differing theoretical orientations in history and the social sciences have long invoked evidence from the Carpathian basin, more specifically, successive institutionalisations of the Hungarian state, to support their approaches. Many have viewed Hungary as the paradigmatic core of Central Europe, sometimes conceptualised as a distinctive ‘third region’ between East and West (Szűcs 1983). For the proponents of world-systems and dependency theories, from the sixteenth century this region of Eastern Europe was ‘semiperiphery’ *vis-à-vis* the core in the West (Wallerstein 1974). It was condemned to backwardness, and liberation from the Ottoman yoke led only to new patterns of uneven development. When industrialisation finally reached Hungary, it resulted in a dual social structure in which the gulf between town and countryside was unusually wide, had significant ethnic colouring, and was not conducive to democratic politics (János 1982; Berend & Csató 2001). The catastrophes of the 1940s led to communist rule and the substitution of plan for market, but the classical Soviet model was flawed by over-centralisation (Kornai 1959). When this had been corrected, it turned out that socialism had more fundamental defects and shortages were inherent (Kornai 1980). Increasing economic difficulties in the 1980s led even sympathetic observers to conclude that the Hungarian combination of plan and market was not ‘feasible’ (Swain 1992). Fast forward a generation and the country has become the leading example of corrupt populism in the form of a ‘mafia state’ (Magyar 2016) or ‘authoritarian capitalism’ (Scheiring 2020a).

This essay analyses data from Hungary to operationalise the heuristic of an academic polymath who was raised in the country’s capital city but spent most of his life in exile. Karl Polanyi (1886–1964) continues to influence many fields of scholarship. Economic anthropologists hail him as the founder of the ‘substantivist’ school.^1^ At the core of his work is a holistic conception that views material livelihoods as embedded in general social relations. This is linked with a modernist notion of rupture, which Polanyi localises in the impact of industrialisation on Britain in the nineteenth century. The ensuing ‘market society’ spawned a variety of countermovements, as the population struggled to protect itself against the disintegrating effects of *laissez faire* markets (Polanyi 1985). The ‘double movement’ of market expansion and societal reactions generated complex interplay, the eventual outcome of which was fascism. As a socialist, the mature Polanyi believed that a ‘great transformation’ was necessary to reintegrate the economy and repair the fabric of society (Polanyi 1985). In this essay I adapt Polanyi’s perspective to the history of Hungary during the last 70 years.^2^ I ground the argument in empirical data from my own field research in particular settlements, while connecting this detail to the national picture.

The global impact of neoliberalism in recent decades invites comparisons with the consequences for Britain and the world of an older liberal ideology in the nineteenth century. Polanyi taught that the very idea of the free market is an illusion, since markets always depend on a range of institutions, first and foremost the state. Subjecting such fundamental goods as land, labour and money to the logic of markets has far-reaching consequences because it disembeds economy from society. According to Polanyi, this was unprecedented in human history prior to the nineteenth century. The victims of the supposedly ‘free’ and ‘self-regulating’ markets respond with countermovements. These range from progressive initiatives to defend workers’ interests (which may result in lower profits for capitalism in the short term but stabilise the system in the long term) to malignant forms of exclusionary politics.

Polanyi’s conceptual apparatus can be applied outside the market-centric paradigm. He defined a countermovement in abstract terms as ‘a reaction against a dislocation which attacked the fabric of society’ (Polanyi 1985, p. 130). I propose to view the repression of political pluralism and markets in the early years of state socialism as such a moment of dislocation, analogous to the rise of the self-regulating market in Victorian Britain.^3^ This Stalinist rupture initiated a dialectic analogous to the double movement of marketisation and societal self-protection under capitalism. Both the centrally planned economy and the ‘paralysed society’ (Hankiss 1990) were modified by reforms from the 1960s onwards. These included economic decentralisation and consumerism in a much more relaxed political climate. The countermovement to Stalinist state socialism later developed further political aspirations, articulated in the contributions of dissidents such as Konrád (1984) and the intellectuals responsible for the *samizdat* journal *Beszélő*. The buzzword that best encapsulated their aspirations was civil society.

Polanyi’s star rose with the advent of neoliberalism, and it was not long before scholars began to apply his insights to the postsocialist victims of ‘shock therapy’ marketisation and propertisation. Less attention was paid to the fate of civil society agendas after 1990, because the triumph of pluralist liberal democracy was long taken for granted. Three decades later, this is no longer the case. Viktor Orbán has had considerable success in imposing a new periodisation, according to which the decisive moment of discontinuity was not the change of system in 1989–1990 but accession to the EU and the formation of his second government in 2010. This representation has much to commend it. Orbán’s decade in power has been characterised in terms of ‘democratic backsliding’ (Cianetti *et al*. 2018; Scheiring 2021) and ‘illiberalism’ (Wilkin 2018; Scheiring 2020b).^4^ My approach interprets the 2010s as a new phase of embeddedness, following an exemplary Polanyian double movement.

The essay is structured as follows. First, I outline the main economic and political-cultural features of the era of embedded socialism in a small town that I have known since the mid-1970s. I then turn to investigate the developments of the first postsocialist decade. In the final substantive section, I explore the rise of illiberal governance in the new century, when the mantle of civil society becomes tarnished and populism becomes entrenched. While I emphasise the material causes of the emotions fuelling this populism, I caution against exaggerated claims concerning the political temperature in provincial Hungary.

Throughout the discussion, like other anthropologists investigating populism, I have to wrestle with a basic dilemma. However distasteful some discourses may be in the eyes of foreign researchers (and of many natives too, of course), and despite the distortions of state-controlled media, my premise is that populist politicians such as Orbán possess democratic legitimacy. His success derives from the fact that, well before he began to tilt the playing field to favour his own party, the appeal and organisation of *Fidesz* were superior to those of all its rivals. My aim is to demystify this appeal, which is so alarming to capital-city liberals, foreign correspondents and EU parliamentarians and officialdom alike. After all, the primary task of anthropologists is to understand and represent the perspectives of their interlocutors. This makes us, in a sense, a populist discipline. Even when we do not agree with the values of those we research, it behoves us to make sense of their worlds by shedding light on the socio-economic factors that shape emotions, discourses and actions, together with the historically embedded symbols and cultural codes on which they feed.

## The state socialist double movement: plan and market

The first decade of socialist rule in Hungary was catastrophic. Political instability culminated in the revolution of 1956 and its brutal repression. Yet just a few years later, in a country in which agriculture was still the most significant branch of the economy, the regime of János Kádár pressed ahead with the mass collectivisation of this sector. In other sectors, the need to correct excessive centralisation was recognised. Economic reforms coincided with an easing of the political climate in the course of the 1960s. A comprehensive package known as the New Economic Mechanism (NEM) was formally introduced in January 1968.^5^ Central planning was not abandoned entirely, but the Hungarian reforms went further in terms of empowering decentralised actors (enterprises and consumers) than measures taken by any other Soviet bloc state. Enterprises were now incentivised to make profits, even if many prices, including the price of labour, were still determined by the state. The result was that factors of production in this socialist simulation of a market were still not deployed as efficiently as in ideal-typical capitalist markets. Low productivity associated with the hoarding of labour was a persistent problem. From the point of view of the consumer, however, the changes were radical and their consequences were appreciated. In addition to improved economic performance by state enterprises and cooperatives, private entrepreneurship flourished both in the form of legally registered small businesses and in the tolerance of a vast range of informal activity ‘beyond the plan’.

The departure from the theory and practice of the Soviet Union was most conspicuous and successful in the agricultural sector (Swain 1985). Both collective and state farms (modelled on the *kolkhoz* and *sovkhoz* respectively) enjoyed considerable freedom as market actors, including the possibility of establishing ancillary enterprises that did not have to be tightly connected with their core farming activities. Outside the new factory-type divisions of labour, members and employees were incentivised to produce commodities for sale on the plots allocated to them by the socialist institution. They did so by drawing on the traditional household divisions of labour. Continuity with presocialist smallholding agriculture was especially strong in regions where scattered vineyards and orchards made it costly to create large fields. In communities such as that of Tázlár, a mere 120 km southeast of Budapest, most farmers were members of a ‘specialist cooperative’ that supported them with supplies of inputs (fertiliser and fodder) as well as marketing services but did not interfere in the internal labour organisation of the household (Hann 1980).

By the time of my doctoral fieldwork in the mid-1970s, historically peripheral, poverty-stricken communities with this type of cooperative were among the most prosperous in the country. They mobilised household members in traditional ways to undertake labour-intensive tasks, such as raising pigs in the backyard, but they now did so within the new socialist market economy. When state slaughterhouses failed to raise purchasing prices in line with villagers’ expectations, household propensity to supply pigs declined. Since maintaining food abundance was of vital importance for the implicit social contract that underpinned ‘goulash communism’, prices were soon raised. As a result, public investments in the improvement of village infrastructure were complemented by unprecedented private accumulation: new houses were equipped with bathrooms, some villagers added a second storey with a balcony, while others prioritised the acquisition of cars and luxury goods. Decades later, I theorised these transformations as the metamorphosis of agrarian backwardness into a new socialist civilisation (Hann 2015).

Social inequalities widened compared with the repressive levelling of the 1950s, but most indicators remained low in international comparison. Since land and the equipment (combine harvesters) needed to farm large fields remained in collective ownership, the new inequalities had little to do with ownership of the means of production. The transformation of rural Hungary in the last decades of socialism can nonetheless be viewed as a form of embourgeoisement, a continuation in socialist conditions of processes incubated in the first half of the century (Szelenyi 1988). In Polanyian terms, villagers led a countermovement against the rigidities of Stalinist central planning. The politics-led disembedding of the 1950s was gradually modified by a new balance of the ‘forms of integration’ (Polanyi 1957). Drawing explicitly on Polanyi’s concepts, Konrád and Szelényi suggested in the 1970s that ‘rational redistribution’ was the basis of a new form of political domination (Konrád & Szelényi 1979). But socialist redistribution was increasingly complemented by market exchange. As the effects of the NEM spread out to affect all sectors of the economy and all domains of social life, a new balance crystallised, based on a comprehensive re-embedding of polity and economy in each other and in society. This balance enabled the inhabitants of marginalised communities such as Tázlár to become better integrated into the national society than ever before.

Tázlár had a population of a little over 2,000 in 1976–1977. It is nowadays well below this figure (official statistics are inadequate due to labour migration). Since the mid-2010s, I have focused more on the nearby market town of Kiskunhalas, where a population in excess of 30,000 in presocialist years has declined considerably since 1990. Since Halas (as the name is commonly abbreviated) was the last town before the sensitive state frontier to Tito’s Yugoslavia, it was home to both Soviet and Hungarian garrisons. The main square in front of the secessionist town hall was renamed after Lenin. Halas had a standard range of socialist institutions, including a large state farm, a modernist office complex that served as the headquarters of the Hungarian Socialist Workers Party (*Magyar Szocialista Munkáspárt*—MSZMP), a new public library and a central Culture House that was accommodated in the town’s former bourgeois *kaszinó*. Yet Halas too experienced the impact of the New Economic Mechanism. Even before its official introduction in 1968, several large enterprises in the capital were instructed by the central planners to establish subsidiaries in Halas. A new industrial estate was created to make this possible, on the other side of the Budapest–Belgrade railway line. Within a few years, thousands of new jobs were created. Thanks to these central measures, by the mid-1980s over one-third of the town’s active labour force was employed in industry.^6^ The demand for industrial workers was so great that it could not be met within urban boundaries, and so villagers were recruited in settlements such as Tázlár. Those who commuted to the new factories by bus did not have to give up their village plots to take advantage of the new wage-labour opportunities. This worker-peasant phenomenon epitomised the economic countermovement to state socialism.

Urban space initially offered less scope to develop the market principle. As argued by Konrád and Szelényi (1979), redistribution was in the hands of a new political class. Some investments benefited the entire population: the massive hospital complex built on a greenfield site in the 1970s served not just the inhabitants of Halas but the rural population of a vast hinterland. But other decisions were inherently discriminatory and led to new inequalities, notably the allocation of apartments in the new housing estates by top officials and the managers of the new industrial enterprises and the state farm.^7^ However, as reforms were steadily extended, new suburban zones for private family houses were designated by the council. Scope for self-interested behaviour and entrepreneurial initiative opened up in many sectors of the economy. The state farm extended its activities in the pharmaceutical sector, while at the same time investing in wineries to produce a range of alcoholic products for foreign (especially Soviet) as well as domestic taste. When foreign investments were encouraged, a large textile factory that had run into economic difficulties was taken over by Levi-Strauss, with the result that, in the final years of the Cold War, ‘American’ jeans were manufactured just a few miles away from the Soviet barracks on the other side of the railway.

The institution known as the ‘enterprise economic work partnership’ was the urban industrial equivalent of the agrarian symbiosis outlined above.^8^ It was widespread by the late 1980s and existed in various forms in Halas. For example, the large state enterprise KUNÉP negotiated contracts with partners in West Germany that allowed it to send highly qualified engineers and surveyors to supervise Hungarian skilled workers undertaking construction projects. Over three decades later, Jakab Merényi, who founded his family firm in 1990 in Bavaria and later became one of Halas’s most successful private businessmen, recalled the experiments of the 1980s positively (Hann 2019c).

Similar initiatives were taken by the Kiskunhalas State Farm, the town’s largest employer, which had long supported its workforce as petty producers and now afforded its managers generous scope for more autonomous economic action. During the last decade of socialism, numerous enterprises and cooperatives in the commercial and services sectors allowed their members to operate more independently as artisans or shopkeepers, incentivised to increase their own incomes by developing new products and improving customer service. Zoltán Krausz recalls that his path to prosperity as a leading retailer of household goods began in the 1970s as an apprentice with a state ironmonger. In the mid-1980s the company allowed him to open a new store on a semi-private (*fél-maszek*) basis. He then opened additional premises to take advantage of the construction boom of the late socialist years. His smooth transition to becoming a fully private entrepreneur after 1990 was facilitated not only by the networks he had established inside Hungary but also by high demand from across the border in Yugoslavia.^9^

Overall, in both town and village we can conclude that, following the initial imposition of socialist institutions ‘from above’, by the 1980s a dynamic countermovement had significantly modified the dominance of planned redistribution. Socialist property relations were not challenged, but new forms of self-interested work and the increasing strength of market signals signified the emergence of a modified socialist economy that warrants the epithet ‘embedded’, in the holistic Polanyian sense. The NEM was a dynamic process and the ways in which it unfolded on the ground, though often criticised, were evaluated positively by most of the inhabitants of Tázlár and Kiskunhalas. However, by the mid-1980s, in the light of foreign indebtedness and the rise of neoliberal ideologies in the West, a new generation of Hungarian economists viewed these compromises with increasing scepticism. Convinced that the role of the state should be reduced to a minimum, and that private property was inherently superior to any form of collective ownership, these young Hayekians urged more radical marketisation coupled with privatisation (Bockman 2011; Kovács 2018; Fabry 2019).

## Civil society as countermovement

Apart from the economic countermovement described above, the disembedding imposed by Stalinist state socialism elicited responses in the spheres of politics and associational life. Given the context of the Cold War and the *realpolitik* demonstrated in 1956 in Hungary and Poland, followed by the events of 1968 in Czechoslovakia, aspirations to a pluralist parliamentary system of liberal democracy were not realistic. Instead, Eastern European dissidents such as Václav Havel, György Konrád and Adam Michnik theorised an alternative mode of opposition to socialist monism: building new alliances in society on the basis of ‘self-organisation’. This chimed in well with international trends in social science, including Western Marxism, where the concept of civil society was being rediscovered and class analysis abandoned. In both West and East it was held that grassroots initiatives and movements, which in the East took the form of countermovements to state socialism, would be the harbingers of more pluralist and emancipatory forms of politics.

But what exactly did the eighteenth-century concept of civil society signify two centuries later? For Gramsci (1971), it was a characteristic of advanced Western states not to be found in the ‘gelatinous’ conditions of Russia. The Eastern European dissidents did not theorise civil society in terms of capitalist embourgeoisement but idealistically, as a revival of liberal bourgeois ideals of freedom. Just as *bürgerliche Gesellschaft* gave way to *Zivilgesellschaft* in German, so *polgári társadalom* made way for *civil társadalom* in Hungarian scholarly literature (Hann 2020, p. 465). For some dissident thinkers, it represented an intermediate realm between the state and the individual (or family). Others envisaged civil society as a third principle of social organisation beyond state and market. Empirical applications concentrated on autonomous associations (Hankiss 1990). Civil society is still routinely operationalised in this way by both Western and Eastern European scholars in the new century. For example, a British scholar and his Russian research partners open a recent study of popular education in Russia with the statement that ‘a civil society is a combination of voluntary civic and social associations and organizations that enables society to function; and which acts as a “shock absorber” between the individual citizen and the state with its monopoly of legal force’ (Morgan *et al*. 2019, p. 1). Neither liberalism nor democracy is mentioned in this definition, yet the congruence with notions of liberal democracy and pluralist parliamentary politics is taken to be self-evident.^10^

Civil society was postulated as a plural agglomeration of decentralised free associations of many types, all based on the principle of self-organisation, and functioning within some form of state (including perhaps a reformed socialist state). In the last decade of the Cold War, however, civil society was also cast as a singular unifying movement, to which an entire citizenry was supposed to subscribe. This second model was obviously incompatible with any version of the socialist state. Some activists did not distinguish these two senses and shifted the emphasis according to context. The collectivist sense triumphed in the emotional fervour of 1989. However, it was never clear how ‘we the people’ sentiments would lead to new forms of pluralist democracy rather than to postsocialist forms of populism.

The traction of elitist intellectual countermovements is often exaggerated in Western accounts of the demise of Eastern European socialism. If civil society was a codeword for ‘liberal democracy’, populations assented readily on the understanding that the demise of socialism would enable their living standards as well as their freedoms to converge with those prevailing in the capitalist West. But even in the Polish case, where mass mobilisation was greatest, it became clear in the unravelling that took place after 1990 that class protest combined with strong sentiments pertaining to the nation and to religion was the most significant motivating force, not the agendas of intellectuals such as Michnik or Jadwiga Staniszkis (Ost 2005; Kalb 2009).

Although the dissidents of the 1980s were concentrated in the major cities (especially capital cities), their critique was not altogether without echoes in smaller settlements. It may have gone unnoticed in villages such as Tázlár, but in Halas the reverberations were considerable. This was due to the fact that the town was selected in 1979 for a pioneering sociological investigation by a team of young social scientists. This research was itself a sign of political relaxation; in the 1950s sociology had been entirely suppressed. The Budapest-based researchers did not content themselves with deferential interviews with local power holders. In addition to such official encounters, they convened public meetings at which residents were encouraged to express their opinions openly. Some cadres became alarmed and accused the researchers of inciting young people to critique the entire system. The research was curtailed and Halas was not even named when the research was written up (A Gergely *et al*. 1986).

This abbreviated research expedition, which served to highlight youth disaffection and socialist institutional atrophy, had a significant impact on the town. Older residents were reminded that their small market town had distinctive *civis* traditions, as a settlement rebuilt from scratch by pious Calvinists from the late seventeenth century onwards, in the wake of the withdrawal of the Ottoman Turks. The peasants of Halas in effect purchased their freedom in 1745, more than a century before the general emancipation of serfs. Stratification in the presocialist era spanned from the rural proletarian to the wealthy ‘peasant-burgher’ (*paraszt-polgár*). The more prosperous strata were Protestant. The Calvinist pastor Áron Szilárdy (1837–1922) became a distinguished orientalist and academician in Budapest. The town’s grammar school (*gimnázium*) was renamed in his honour. In the course of the nineteenth century, however, Protestants were outnumbered by Catholic immigrants who colonised the town’s vast uninhabited hinterland, the *puszta*. Apart from church associations, many residents also belonged to guild-like bodies and clubs. These were independent of the state and supported by the contributions of their members. Almost all of these bodies were suppressed in the early years of socialism (Miheller 2019). The Budapest research project was a catalyst for some citizens of socialist Halas to make a concrete connection between the civil society rhetoric of dissident intellectuals in the capital and the history of their market town.

No one was more excited by these developments than László Lukács, aged 16 and a *gimnázium* pupil when the Budapest researchers arrived. The descendant of a *paraszt-polgári* family himself, he was recruited a few years later by the local cultural bureaucracy to work at the municipal cinema. As the political climate relaxed across the country, with the help of a few respected local intellectuals, Lukács and a few friends applied in September 1988 to establish an association called the Urban Youth Workshop (*Városi Ifjúsági Műhely*—VIM). After a few bureaucratic hurdles, permission was granted. The VIM was experienced by its enthusiastic members as the first stirrings of a free civil society. The association was nominally non-political. It organised summer camps with a focus on literature, including the work of Budapest dissidents. Culturally, László Lukács was a conservative, keen to revive the *civis* traditions of his town. In the free parliamentary elections of 1990, he campaigned on behalf of the Hungarian Democratic Forum (*Magyar Demokrata Fórum—*MDF), which won the elections nationwide. Under Lukács’s leadership, the VIM went on to spawn a number of specialised groupings to cultivate local and national heritage. He had the know-how to obtain grants (for example, from the Open Society Foundation of George Soros) and to produce newsletters and magazines, in which the terms *polgári* (bourgeois) and *civil* were deployed as synonyms to propagate the concept of a civil society to wider audiences.

But the MDF did not triumph in Halas. In the late 1980s other local activists were already in touch with well-known national dissidents, by now organised as the Alliance of Free Democrats (*Szabad Demokraták Szövetsége—*SZDSZ). Zoltán Tóth had participated personally in their activities since his studies of horticulture in the capital in the 1970s. He was the prime mover in establishing the SZDSZ in Halas, managing to raise support from local entrepreneurs whose interests, he argued, would be promoted by the new party. Tóth and his allies launched a journal which they called *Contrast* (*Ellentét*). Articles in 1989–1990 invoked the concept of civil society in much the same way as the pamphlets and flyers of László Lukács. The main difference was that the SZDSZ activists did not attach the same weight to local traditions and culture. In Halas, the SZDSZ out-performed the MDF, both in the parliamentary elections and in the local elections a few months later. Zoltán Tóth was elected as the first postsocialist mayor of the town, at the head of an SZDSZ–*Fidesz* coalition. It seemed that the prospect of a future-oriented liberal reconstruction of economy and society had triumphed over a conservative appeal to reconstitute the nation on the basis of its cultural heritage. The local elections of October 1990 represented the zenith of the liberal intellectual countermovement in Halas.

## Economy, politics and civil society in the 1990s

In Halas as in the rest of the country, both agricultural and industrial sectors experienced massive dislocation in the early 1990s. The privatisations included the region’s largest construction company KUNÉP (mentioned above) as well as the state farm. The latter enterprise had been struggling for years due to difficulties in marketing its showcase products, following the impact of Mikhail Gorbachev’s anti-alcohol policy. The unskilled were invariably the first to be made redundant. Virtually the entire Roma community, after decades of struggling to adjust to the socialist compulsion to work, now found itself unemployed and dependent on state assistance.^11^

The economic problems of Halas were accentuated by the closure of the Soviet barracks and by the instability created by civil war in nearby Yugoslavia. At the same time, each of these factors opened up plentiful black market economic opportunities. Unemployment, previously non-existent, rose to 12.5% by 1992 and the industrial workforce halved (Hann 2019b, p. 553). It was possible to interpret the collapse of socialist industry in Halas as confirmation that both the initial central decision in the mid-1960s to establish large enterprises on a new industrial estate and the general principles of the NEM were flawed (Végső 2019). That view is not shared by many of those who experienced those shocks. The managers who salvaged something from the bankrupt enterprises, typically re-employing only a small fraction of the former workforce, condemned the failure of the state to offer economic lifelines. Jakab Merényi, mentioned above, who succeeded in creating a highly successful company out of the economic work partnership that he had initiated within KUNÉP, deplored the fact that most of the plant and workforce of this socialist enterprise were simply abandoned by the MDF-led government in the early 1990s.^12^ The headquarters of KUNÉP were acquired by a Tázlár entrepreneur who, in the space of a few years, transitioned from village locksmith to major player in the country’s newly liberalised tobacco market. His methods were likened to those of a Mafia gangster by the SZDSZ mayor of Halas, Zoltán Tóth.^13^ To the general population they exemplified the way to prosper in the years of ‘wild capitalism’ (*vad kapitalizmus*).

Jakab’s views are shared by other ‘winners’ of the transformation, namely, former socialist managers who managed to build up private businesses after 1990. János Gidai was a young engineer at the Halas subsidiary of Ganz, a Budapest enterprise best known internationally for manufacturing trains. When this enterprise ran into financial difficulties in the late 1980s, the ministerial solution was to privatise and dismember the conglomerate to attract foreign capital. After a short period in British ownership, the core business was sold to an Austrian firm, which (according to János) like most other foreign investors was only interested in mopping up competition. The Halas subsidiary was divided into four units for privatisation. János and 26 colleagues took over compressor production, and soon succeeded in developing new products and opening new markets. But after buying out his partners and almost three decades of expansion, the workforce failed to reach three figures, compared with the 800 who had worked at this enterprise in socialist days. What riled János most when he looked back was that the lack of state support at the time of transition had led to the liquidation of Hungarian firms whose products were superior in quality to those of the foreign competitors who proceeded to take over domestic markets. He deplored the fact that the old system of industrial apprenticeships disappeared with the factories, such that, despite unemployment, it has become difficult to recruit skilled workers in Halas. Nowadays, János said plaintively at the end of our conversation, even college graduates opt for dishwasher jobs in London: they may earn more money that way, but what about their futures, he asked, and the future of their town?^14^

The uncertainties of the first postsocialist decade were reflected in the results of parliamentary elections. In 1990 the Kiskunhalas constituency elected László Horváth, an elderly veterinarian, the candidate of the Independent Smallholders’ Party (in full, the Independent Smallholders, Agrarian Workers and Bourgeois/Civic Party; *Független Kisgazda, Földmunkás-és Polgári Párt*, generally abbreviated to *Független Kisgazdapárt*). In 1994 it returned László Varnai, a socialist (and ex-communist) who had formerly worked as a legal counsel at the state farm. In 1998 it elected a lawyer who had defected from the SZDSZ to *Fidesz*. Zoltán Tóth served as mayor throughout these years, but he was repeatedly obliged to change his coalition partners. Management of the municipality was hindered by the fact that the local coalitions never coincided with those governing in the capital. The mayor and most of his supporters quit the SZDSZ when they judged that the intellectuals who had founded this party had no interest in the issues that mattered in the provinces, in particular assuring jobs and livelihoods (Hann 2019c). Tóth founded a new association, the Urban Civic Circle (*Városi Polgári Kör*—VPK). According to its constitution, VPK members were required to distance themselves from all political parties. Although it never numbered more than a few dozen members, this association remained a significant force in local politics in Halas until 2002.

How did economic disintegration and political instability affect developments in culture and civil society? In the early 1990s, undismayed by the disappointing local performance of the MDF in the elections of 1990, László Lukács continued to work at the cinema and to promote cultural activism in the VIM. When the directorship of the municipal Culture House was advertised in 1994, his application was successful. He held this post for the next five years, eventually changing the name of its headquarters to Communities House (*Közösségi Ház*). During this period, the socialist-led government of Gyula Horn introduced new regulations for civil associations and their funding.^15^ The ostensible goal was to place the funding of such bodies on a more secure basis by allowing taxpayers to donate 1% of their payments to bodies that were recognised to operate in the public good. The effect of this measure, strongly criticised by activists such as Lukács, was to draw a clear line between associational activities and politics. Political groupings were not eligible for support. This contradicted Lukács’s vision of generating new forms of politics at the local level that would permit civil society to displace the moribund institutions of national party politics. He commented publicly on the new laws as follows:

The situation is that the elected representatives of the political power want to push the self-realisation of civil society (*civil társadalom*) back to the level of a club movement, and the middle classes to the status of mass-consumption citizens with no means to realise their interests! (Lukács 1998, p. 1)

It is clear here that, although he has adapted *civil* in place of *polgári*, for Lukács the activist, the programme of civil society is concerned not so much with the emancipation of the whole of society as with the interests of a particular bourgeois class. Tensions arose between Zoltán Tóth in the Town Hall and László Lukács at the Culture House. When the directorship of the latter came up for renewal in 1999, the liberal mayor opted to appoint the previous socialist director. Lukács had to seek work elsewhere.

By the end of the first postsocialist decade, associational life in this small town had indeed contracted again. The VIM, along with other newly established associations that were meant to express postsocialist freedoms, struggled to maintain support and folded. Jakab Merényi co-founded a new club for entrepreneurs and another was created for the unemployed, but neither established a regular rhythm of meetings. Most respondents I encountered during my fieldwork felt that cultural standards had been higher in the socialist decades, when more people went to the cinema and the quality of the films was superior. Those who were young in the Halas of the 1990s are more likely to recall a noisy disco culture in private clubs and bars than participation in events organised by the new independent associations. Ironically, those who associated civil society with bourgeois habits of earnest debate and cultivation of the arts had to concede that these objectives had been much better met within the framework of the socialist Culture Houses, long scorned as the antithesis of civil society (Miheller 2019).

With several dozen municipal employees responsible for local television, numerous seasonal festivals and a calendar of events that would be impressive in comparison with towns of comparable size anywhere in Europe, the *Kiskunhalas Almanach* published at the turn of the millennium conveys a rather positive image of postsocialist civil society (Szakál 2002). In total, 95 associations and foundations are listed, in addition to separate listings of churches, political parties and ‘social organisations’.^16^ But by the end of the first postsocialist decade the proportion of the population playing any active part in these associations and cultural events was in fact quite modest (Miheller 2019).^17^ The emergence of a new bourgeois elite was symbolised by the formation of a Rotary Club in 2009. However, for the majority of postsocialist citizens, struggling to cope with existential worries, clubs and associations were an irrelevance. The quality of their civil society, which had spurred youngsters like László Lukács to action in the 1980s, was no longer a priority. Young people were more concerned with how to obtain the resources to build or buy a house. Even before Hungary’s accession to the EU in 2004, many were looking for work in Western countries, even if this meant abandoning the career for which they had gained qualifications. I return to these trends below.

## Illiberal democracy as a countermovement

The cohort of liberal intellectuals most prominently committed to the agenda of civil society in the 1980s had generally departed from the national political stage by the end of the century. The major exception was Viktor Orbán, who rebranded his *Fidesz* party as the *Magyar Polgári Párt* as part of his strategy to garner the votes of those who had hitherto supported the MDF and the Smallholders.^18^ Orbán became prime minister in 1998, but he was defeated by the Hungarian Socialist Party (*Magyar Szocialista Párt*, successor to MSZMP) in 2002 and again in 2006—on the latter occasion by a young politician with a comparable lust for power, Ferenc Gyurcsány. But the welfare promises made by the socialists could not be kept.^19^ EU accession in 2004 brought little relief, and the 2008 global financial crisis further accentuated the troubles. Orbán’s response to electoral defeat in 2002 defined the new epoch. His pithy declaration that ‘the nation cannot be in the opposition’ encapsulated the populist challenge to pluralist liberal democracy. The nationwide network of ‘civic circles’ that he organised in those years can be theorised as a right-wing Gramscian programme to gain power by first capturing the *società civile*. Ethnographers have shown that these grassroots associations contributed significantly to the consolidation of *Fidesz* in these years. Unlike the civic circle established by Zoltán Tóth in Kiskunhalas, whose members were precluded from party political activities, the activists of Orbán’s circles campaigned to present one political party as the true representative of the people, namely, of the entire Hungarian nation (Halmai 2011). Orbán’s populism acquired an appropriate designation in 2014 when he himself coined the notion of ‘illiberal democracy’.^20^

I interpret this Hungarian trajectory as a reaction to the privatisation and marketisation of the 1990s, which set the entire region on a path analogous to that pioneered by liberal Britain in the nineteenth century, as definitively analysed by Karl Polanyi. In Kiskunhalas, economic indicators remained unfavourable in the first decade of the new century. After two decades in which it was consistently one of the town’s most popular employers, especially among women, the Levi-Strauss factory was liquidated at short notice in 2009. Production was transferred to a plant in Romania where labour costs were lower (Kohout 2009, pp. 1–2).^21^ In the same year, Daimler-Benz began a massive investment to build a new car factory at the county capital, Kecskemét (Jacobs 2017, pp. 265–69).^22^ The jobs available here, while well-paid by local standards, involve a commute of roughly an hour in each direction. There is resentment that the wages paid for monotonous work at the conveyor belts are at best one-third of the wages paid for carrying out the same work at Daimler-Benz factories in Germany. Meanwhile in Hungarian factories, including some that flourish in the supply chains of the multinational corporations, the majority of workers receive only the minimum wage, supplemented, if they work well, by an informal bonus payment that is not declared for taxation purposes and does not count towards pension entitlements. This practice is common in Halas; the low rate of taxation under Orbán’s governments has had little or no impact in this regard.^23^

Departing from his earlier endorsement of the market economy, from the moment of his re-election in 2010 Viktor Orbán began to intervene very actively in market mechanisms. In Polanyian theory, land, labour and money are the three ‘fictitious commodities’: goods that are not produced to be bought and sold but need to remain embedded in systems of social control (Polanyi 1985). The *Fidesz*-led government renationalised significant tracts of land, built up the largest workfare schemes in Europe, and was not afraid to take on international banks and deviate from internationally prescribed neoliberal monetary policies. Millions benefited from government measures to reduce utility bills. Following accession to the EU in 2004, rural households began to enjoy the subsidies of the Common Agricultural Policy. During the 2010s these payments were commonly attributed by farmers to Orbán’s resolute policies in standing up to Brussels and fighting to defend Hungarian entitlements (Kürti 2020, pp. 65–6). It was widely documented that the prime beneficiaries of this economic countermovement to market logic were family members and party cronies of the prime minister (Magyar 2016). But many citizens considered this unavoidable—previous governments had been no better in this respect—and at least under *Fidesz*, through measures such as housebuilding subsidies, the reduction in utility bills or the distribution of benefits in kind such as winter fuel, ordinary Hungarian families were receiving some protection from the gales of global markets. A prominent example of the countermovement against neoliberal economic policies in Kiskunhalas was the action taken to secure the future of the Semmelweiss hospital complex, which had been effectively privatised under an earlier socialist-led government.^24^

Political developments in Halas in the new century were complex. When Zoltán Tóth decided in 2002 not to run again for the mayorship, the civic association that he had founded disintegrated. For the next eight years the town hall in Halas was headed by the socialist László Varnai, mentioned above, who had represented the town in parliament between 1994 and 1998. *Fidesz* out-polled the socialists in the municipal elections of 2006, but this former communist was personally popular and he continued as mayor with the support of smaller parties and independents. With the MDF no longer a significant force either nationally or locally, the former cultural activist Lukács joined *Fidesz* and was elected as a councillor on their ticket in 2006. He was elected to parliament in 2010. However, in the local elections of that same year, he failed to become mayor. While *Fidesz* now dominated the council, the candidacy of Lukács was undermined by rivals within his own party, which allowed another former communist to obtain the top position. Lukács experienced further disappointment in 2014 when, following a contraction in the size of the parliament and a re-drawing of constituency boundaries, he was obliged to give up his safe parliamentary seat to a *Fidesz* colleague with a longer record of service to the party. By this time, *Fidesz* had identified an alternative mayoral candidate, young and educated in economics. Although not natives of Halas, the young man’s family had built up a strong entrepreneurial profile after 1990. Thus in 2014, rather later than elsewhere, the town’s political floundering came to an end: at European, national and local levels, only one party counted. *Fidesz* had no trouble in repeating these victories in 2018–2019.^25^ Over the previous decade the socialist vote had collapsed.^26^ The strongest opposition in the 2010s, here as throughout the Hungarian provinces, came from the extreme right, initially *Jobbik* and, later, *Mi Hazánk* (see Szombati 2018).

Contrary to the images propagated in foreign media coverage of Hungary (shaped by the extremes of the state media and by *causes célèbres* such as Orbán’s vendetta against the Central European University), the political temperature in this provincial town in the 2010s remained low. It did not rise significantly during local election campaigns. Even in more keenly fought national campaigns, turnout never rose above two-thirds. The young mayor and the member of parliament are not considered ideologues. They are perceived by my fieldwork interlocutors to be working quietly and pragmatically on behalf of their constituents. Apart from poster campaigns ahead of a referendum, the new one-party system is not omnipresent in everyday life. Those who suggest comparisons with Stalinism in the early 1950s are wide of the mark. While the present system undoubtedly has authoritarian aspects (Fabry 2019; Scheiring 2020a), at the grassroots the populist response to neoliberal marketisation takes more mundane and even humane forms.^27^ My fieldwork interlocutors spoke of rumours about municipal contracts being awarded exclusively to loyal *Fidesz* supporters, but similar stories were told under previous mayors and most Halas citizens agree that their present representatives are competent and ‘normal’, in comparison with the *Fidesz* hardliners who dominate the national stage. Local television is more restrained than the state media. While the profile of the major churches in the latter is high, my fieldwork in Kiskunhalas suggests that local clergy do not preach politics from the pulpit and church attendance has not increased significantly since 1990.

The *Fidesz* mayor who took office in 2014 expanded the workfare programme initiated by his socialist predecessor. Although the jobs allocated to participants often seemed rather pointless, the scheme was welcomed on the moral grounds that no one should receive state benefits without working (Hann 2018).^28^ Workfare was strongly supported by the Roma deputy mayor, who was pleased that, after two decades of passive welfare dependence, members of his community were learning the disciplinary value of labour and thereby being reintegrated into the local community. This individual employs many co-ethnics in his own successful small enterprises in the construction and waste disposal sectors. It is widely assumed among my respondents that this deputy mayor mobilises the Roma vote for *Fidesz*; other entrepreneurs who receive contracts from the town hall are said to do the same.

Apart from the large Daimler-Benz factory in Kecskemét, economic options remain limited. Local politicians and businessmen have pinned their hopes for attracting investors on an upgrading of the Budapest–Belgrade railway connection within the frame of China’s ‘Belt and Road’ scheme. But even if this comes to fruition, it is by no means certain that it will bring new industrial investments to Halas. Following the ‘migration crisis’ of 2015, new jobs were created in connection with the intensified security measures along the nearby frontier. However, labour migration abroad remains high, especially among qualified youth. The only significant foreign investment has come in the supermarket sector: Tesco, Aldi and Lidl compete for custom in a town that nowadays has fewer than 30,000 inhabitants. They do so in large stores remote from the historic centre. Spar has a few outlets in the centre, but streets that used to thrive in socialist days are now empty. Several shops are rented by Chinese entrepreneurs who retail bottom-of-the-range goods. Only the second-hand sector holds up well. The welfare cuts in the 2010s have forced large segments of the population to use *diszkont* shops and to borrow money, either from banks or from more flexible lenders such as *Provident*, in order to meet urgent needs.^29^

The family businesses that I have investigated in recent years have experienced very different trajectories. Jakab Merényi, whose construction empire emerged from an enterprise economic work partnership in the 1980s, has recently handed over the reins to a new generation. The firm operates nationwide, with multiple provincial retail sites and offices in Budapest and Győr supplementing the headquarters in Halas. Whereas Jakab is content that his company is now registered on the stock exchange as a private company, with responsibilities and profits shared according to a ‘family constitution’, János Gidai has had more difficulty in meeting the challenge of succession.^30^ Lacking a direct heir, he groomed a suitably qualified nephew to take over. However, János is critical of the younger man’s inclination to maximise short-term profits and worries about the future of the firm that he carefully built up from the wreckage of a socialist enterprise.^31^ As for Zoltán Krausz, he has bucked the trend by continuing to respect collective norms in his retail store, which depends on a stable, brigade-like group of trained employees. But he cannot sell household goods as cheaply as the chains in larger cities, and online shopping is an additional threat. Since Zoltán’s two sons have long moved away from Halas (one to Budapest and the other to Scandinavia), the future of his business is uncertain.^32^

Overall, economic developments are not conducive to a good *Stimmung* in the town. There is nothing like the ebullient embourgeoisement that I documented in my research during the last decades of socialism. I nonetheless suggest that *Fidesz*’s domination in the 2010s represents a phase of re-embedding, following the political, economic and social turmoil of the preceding two decades. In the terms of Polanyi (1957), market exchange as a ‘form of integration’ has been modified by more active state interference since 2010. The mechanisms of redistribution have changed considerably. While overall public spending on health and education has declined, strategic subsidies give the impression of a caring government. Meanwhile a new national bourgeoisie benefits from opaque mechanisms to channel transfer income from Brussels (Magyar 2016; Scheiring 2021).

Although the new embeddedness means stability, which in principle citizens welcome, as a result of the general socio-economic decline most residents of Halas I encountered would agree that interest and participation in the public life of their town have waned. The general sense of decline influences the private decisions of those in a position to make choices, such as whether or not to build a new house, or to hold a lavish wedding reception. The trend is reflected in the declining standards of the town’s football team, which, despite the recruitment of professional players, has failed to attract more than a handful of spectators in recent years.^33^ The reputation which the town enjoyed across the country for its lively youth culture had faded even before the end of the first postsocialist decade and has not improved since. On paper, Halas has a packed calendar of events, but few attract crowds.^34^

It is against this background of grey everyday life and lack of opportunities that illiberal populism has taken root. Clientelist politics are important, but so too are an aggressive right-wing popular culture and performative dimensions of everyday nationalism, for example, in the fanning of anti-Roma sentiment (Feischmidt 2014). In the absence of a fervent party membership, effervescence is experienced vicariously through posters and the media. The ritual calendar has been transformed. Irredentist nostalgia for the ‘great Hungarian kingdom’ that was dismembered by the victorious powers at the Treaty of Trianon in 1920 permeates school textbooks and the public sphere. In Halas, as throughout the country, concrete commemorations of the centenary of the Trianon catastrophe in 2020 were thwarted by the COVID-19 pandemic, but the symbolism is ubiquitous online.

By far the most significant event of recent years in terms of political imaginaries was the ‘migration crisis’ of 2015, when it is estimated that as many as one million refugees and migrants passed through Hungary. By this time, local Roma had been disciplined by workfare and reintegrated into the hierarchy of Hungarian society as its bottom stratum. Some migrants originating in Syria, Afghanistan or North Africa were perceived by locals to have a vague physical resemblance to local Roma. In any case, they quickly came to constitute a new ‘other’ for the Hungarian majority,^35^ and therefore grist to the populist mill. At the height of the crisis, significant numbers of migrants received temporary accommodation at a former barracks on the outskirts of Kiskunhalas. Some of my respondents noted having seen them in the nearby Tesco store and a few ventured as far as the town centre. Rumours of misdemeanours were widespread, but few residents of Halas ever set eyes on persons classified by their government as ‘illegal immigrants’. Images and opinions were entirely formed through the national media campaigns. If these foreigners travelled by taxi and evidently had superior mobile phones to those of provincial Hungarians, how could they be deserving of support? It was understood that few migrants had any desire to stay in Hungary, but few of my interlocutors in Halas thought that they should they be allowed free passage to Germany, where they would be welcomed, integrated into labour markets, and soon be able to earn higher wages than disadvantaged Hungarians.

The migration scenario of 2015 and its continued exploitation in the state-controlled media over the following five years helps us to answer a puzzle raised by Ernest Gellner in the 1960s concerning the routinisation of populist power. Gellner queried ‘a definition which entails that a successful populist cannot be called a populist’.^36^ The solution lies in consolidating populism into full-fledged illiberalism. This is most easily accomplished *via* the specification of new external enemies and ratcheting up the patriotic rhetoric, accompanied by injections of demographic delirium and Islamophobia. This appears to work: the great majority of provincial Hungarians support their prime minister when he rejects the allocation of quotas of refugees by Brussels.^37^ Citizens of Halas I spoke to who recalled the summer of 2015 considered that the German chancellor, Angela Merkel, had made a decision that violated international law. It was hypocritical on her part, they claimed, to demand EU solidarity for a cause to which they have never assented. Viktor Orbán was generally applauded for defending the values of European Christian civilisation against a degenerate liberalism that has infiltrated Western political parties, even those calling themselves Christian Democratic. The Hungarian media ceaselessly target George Soros, who is said to fund most of the ‘pseudo-civic organisations’ undermining Hungary and Europe.^38^ Smear campaigns against opposition politicians did not prevent some of the most bitterly denigrated critics of the government from being elected to mayoral posts in the local elections of October 2019 (Novak 2019). This glimmer of hope for the opposition was renewed at the end of 2020 when key opposition leaders pledged to unite behind joint lists of candidates in the parliamentary elections scheduled for spring 2022 (Bayer 2020). But in the meantime, no civil atmosphere for political debate exists at any level. Opposition leaders are known only through the media: they do not visit provincial towns such as Halas and are somehow ‘not real’ (Kürti 2020, p. 67). By contrast, the benefits of illiberal democracy are tangible. Unlike their opponents, Viktor Orbán and other national leaders of *Fidesz* have taken the trouble to visit Halas on numerous occasions.

In the liberal media across the ‘old EU’, perhaps especially in Germany, the dominant perception of the Visegrád Group, and of Hungary in particular, can be summarised as abhorrence. Critics like to point out that this new member should be grateful for the support it gets from Brussels (and thus indirectly from German taxpayers) and has no business subverting the edifice with irresponsible populist rhetoric. It is commonly suggested that one needs to investigate long-term history, not just the socialist experience but also the preceding centuries of economic and socio-cultural backwardness on the margins of Europe, to understand why the Magyars are incapable of implementing what enlightened Westerners have been implementing since at least the eighteenth century.^39^ But this moral shaming by means of culturalist explanations is unconvincing, if only because similar expressions of discontent are increasingly common all over the world as globalisation accelerates. No explanation of today’s illiberal democracy can ignore the legacies of Hungary’s imperial past and the relative successes of the late socialist era, especially in the countryside. But alongside memory politics, emotions and symbols, it is equally important to investigate contemporary political economy, for example, by comparing the transfers extracted from Hungary by transnational capital with those entering the country in the form of cohesion funds from Brussels (Piketty 2018).

## Counter-countermovements

The countermovement to neoliberal marketisation that currently appears so strong throughout provincial Hungary has itself generated powerful reactions. Civil society remains a prominent concept. In the new century it has become ever more important to take account of new forms of transnational connectivity. Since 1990, postsocialist investors driven by calculations of profit have been joined by legions of private philanthropists, the representatives of international aid agencies and civil servants of Western states disbursing know-how to the civilisational newcomers. In these circumstances, especially in the capital cities, novel forms of ‘global civil society’ (Kaldor 2003) have emerged, dominated by NGOs whose staff communicate in English and whose major priorities are to secure funding to support the next short-lived project for civil society support. The rise of NGO discourses and their salience in so many fields evacuated in whole or in part by state agencies has been theorised in terms of a ‘second renaissance’ of civil society (Jensen & Miszlivetz 2006). But, as these authors go on to observe, these forms of NGO activity can also be deeply alienating and destructive of social cohesion (cf Wedel 1998; Sampson 2002).

Global civil society for Kaldor (2003) is a multi-scalar phenomenon in which transnational movements and international organisations that subscribe to a universal language of human rights are prominent, while locally rooted initiatives have a role to play if they endorse the broader frames of reference. But the latter are missing in provincial Hungary. Populist leaders claiming to be the true representatives of Hungarian society will deride bodies concentrated in the capital city, which are often dependent on foreign sources for at least part of their funding. When such NGOs take up political positions that criticise or contradict the programmes of the democratically elected government, it is only to be expected that ruling politicians will condemn pseudo-civic organisations, alleged to be out of kilter with the needs and aspirations of the national community. These ‘civil society effects’ (Walton 2019; see also Hann 2004) give rise to spirals of illiberalism and raise fundamental issues concerning parliamentary democracy. The Liberal counter-countermovement, though of considerable significance at the national and the European levels, has no resonance in Kiskunhalas. When NGO humanitarian aid for ‘illegal migrants’ was criminalised by legislation in June 2018, few provincial Hungarians could understand how this could possibly be morally controversial.^40^ To the extent that these citizens are aware of an ethically grounded liberal critique of Orbánism, it is commonly dismissed as ill-informed posturing by those whose own positions are incomparably more privileged than the situation of ordinary Hungarians.

## Conclusion: neoliberalism and illiberalism

George Schöpflin (2016) has argued that Orbán’s invocation of ‘illiberal’ in 2014 refers solely to his rejection of neoliberal economic principles. Clearly there is more to it than this. Populist leaders in postsocialist Eastern Europe invoke national and Christian values to counter those of liberal cosmopolitanism. They are accused by Western social scientists of interfering with the judiciary, the mass media and established academic institutions. The catch-all terms for these phenomena are ‘democratic backsliding’ and ‘illiberal democracy’. Hungary is the paradigmatic case, and there is much discussion of Orbánism (Kürti 2020) and Orbánisation (Wilkin 2018). But Schöpflin himself was a professional social scientist before serving four terms as a *Fidesz* representative in the European Parliament. He had a ‘ringside seat’ (Schöpflin 2021, p. 190) at the increasingly fractious battles between liberal ‘old EU’ and the postsocialist newcomers, especially the four states of the Visegrád Group. His new book probes both the historical background and the contemporary *realpolitik* within the EU that has brought systemic conflict between patriotic Christian conservatives in the East and increasingly punitive cosmopolitan liberals in the West (Schöpflin 2021). Schöpflin offers a provocative alternative to the denunciatory consensus of the social science literature and his analysis can help the ethnographer to make sense of the life worlds of provincial Hungarians.

In Hungary, the coarsening of the political culture sank to new depths following the migration crisis of 2015, when the fence constructed by Orbán along the Serbian border became the prime symbol of national and even civilisational pride. Muslims were now the ‘other’ to be excluded from Christian Europe. The European Commission in Brussels, in the person of Jean-Claude Juncker, and the German government in Berlin, in the person of Angela Merkel, were accused in a daily barrage by government-controlled media of abandoning their duties. The media attacks claimed that these European leaders purported to represent Christian parties, yet in reality, aided and abetted by the Jewish financier George Soros, were pursuing policies that were endangering the civilisational integrity of the continent (Bayer 2019).^41^

Focusing on the market town of Kiskunhalas, in this essay I have explored the socialist market economy, liberal civil society and illiberal democracy as three complementary kinds of countermovement. This involved applying Karl Polanyi’s heuristic to the dislocation caused by high socialism. The socialist market economy was, to a large extent, shaped from below, in both agricultural and industrial sectors. Civil society was a reaction to monolithic socialist rule on the part of an oppositional cultural intelligentsia. Illiberal democracy is a more orthodox Polanyian countermovement, a genuinely popular reaction to the ‘market society’ that replaced socialism after 1989. I have argued that the intellectual ideals of a free and democratic civil society as articulated before 1989 foundered thereafter in the face of a political economy that precluded such ideals. Instead, the impact of neoliberalism engendered illiberalism.

The populist politicians who came to exercise a near monopoly of power in the V4 in the 2010s can no longer be classified as a countermovement. They have become the new establishment. The power of *Fidesz* is so deeply entrenched in the Hungarian provinces that it is reasonable to diagnose a new one-party system. The question is how long leaders such as Orbán can maintain their image as populist anti-establishment rebels as they consolidate their hold on power. The political opposition has been hitherto fragmented and ineffectual. Criticism comes, as it did under socialism, primarily from intellectuals, who are nowadays likely to work with urban NGOs. But the new incarnations of an earlier liberal countermovement are stigmatised for undermining the national cause, and it is therefore hard to challenge today’s rulers. Unlike their socialist predecessors, they have electoral legitimacy.

Civil society promotion cannot compensate citizens for the massive disruption of livelihoods and security. This conclusion is valid for many other postsocialist states—certainly for all members of the Visegrád Group. The extreme nature of the Hungarian case derives from the fact that the sense of precarity and relative deprivation is greater in a country where so many households, especially in the countryside and small towns, participated in dynamic processes of accumulation in the last decades of socialism. These citizens have experienced an overwhelming sense of loss of control, for which the governments of the first postsocialist decades are held responsible. Those elites failed to take care of their constituencies, the people. Efforts to promote a civil society have instead functioned to widen social differences, especially the old gulf between *urbanus* and *ruralis*. ‘Talking civil society’ (Jensen & Miszlivetz 2006, p. 143) has spawned a plethora of organisations that populist politicians no longer try to co-opt but instead brand as ‘pseudo-civic’, on the grounds that they undermine collective national interests. Left-liberal elites are held to be pursuing their selfish interests and failing to protect their societies. This protection can only be assured by populist re-embedding.

Despite the scorn expressed towards Brussels, there is no question of withdrawal from the union that has provided substantial material aid to the region in the last 15 years and done much to buttress the emergence of a domestic capitalist class. But transfers received from Brussels through so-called cohesion policies are insufficient to close the historic gap. Provincial Hungarians do not perceive or experience any convergence with the ‘old EU’. Populist politicians pursue policies that address the claims and complaints of these citizens, emotional as well as material. In the relative economic stability of the 2010s, they were perceived to be telling the truth about power, in particular, its abuse by cosmopolitan elites. Policies that temper the impact of hyper-globalisation by a return to work-based redistribution in the interests of maintaining equality, well-being and the cohesion of the nation as a solidary community, have electoral appeal. But the low turnout at elections suggests that the noisy illiberalism that dominates the public sphere may not in fact have such deep roots in society. Support for illiberal populists is hardest to explain where it can be demonstrated that the commitment to redistribution grounded in need is purely cosmetic, since their economic policies in reality serve the interests of a new class and widen inequalities. But this too is a familiar act of deception in the double movement of Karl Polanyi’s ‘market society’.

## Footnotes

1 See Dale (2010, 2016).

2 See also Hann (2019a). Polanyi’s attachments to Hungary ran deep, but he did not investigate Hungarian economic and social history in the same depth that he studied Britain, West Africa and Ancient Greece.

3 In a long-term perspective, Marxist–Leninist socialism can itself be viewed as a spinoff of capitalist industrialisation in Western Europe. For my purposes in this essay, the extension of Polanyi’s schema to analyse a politics-led disembedding is justified by the specific ways in which Hungarian socialism was imposed by an external power.

4 See also Scheiring, this issue.

5 Swain (1992, pp. 85–113) gives a good overview of the evolution of these reforms. See also Hare *et al*. (1981). The NEM was the subject of a summer university in which I participated in Budapest in August 1974. However, I learned more in visits to teeming marketplaces, shops and restaurants than in the classrooms of the Karl Marx University of Economics.

6 The proportion was higher than that achieved in the county capital Kecskemét, although the latter had experienced significant agriculture-related factory development in the presocialist decades; see Ván (1985).

7 See Szelenyi (1983).

8 Radically different production processes prevented close replication. Swain (1992, p. 178) is critical of the ‘surreal’ nature of the enterprise partnership, the introduction of which enabled modest improvements in overall performance, but only by encouraging individuals to adopt a cynical stance towards work and property in the collective sector. Similar reservations were expressed by many domestic critics.

9 Author’s conversation, Kiskunhalas, 9 September 2019.

10 For further discussion, see Hann (1990, 2020), Kumar (1993), Outhwaite and Ray (2005), Wagner (2006).

11 See Szombati, this issue.

12 Author’s conversations, 5 September 2018 and 18 September 2019.

13 Author’s conversation, 10 September 2019, confirming statements disseminated in local media in the early 1990s.

14 Author’s conversation, 9 September 2019.

15 This analysis is based on a conversation with László Lukács on 20 September 2019 and later correspondence, during which he repeated the accounts given in journalistic contributions two decades earlier. For the legislative reforms, see: ‘1997. évi CLVI. Törvény a közhasznú szervezetekről’, available at: https://mkogy.jogtar.hu/jogszabaly?docid=99700156.TV, accessed 6 October 2021.

16 The *Kiskunhalas Almanach* classifies the VPK under ‘parties, party organisations’, though it is noted that members ‘may not utilise the association for any party-political purposes’ (Gyarmati 2002, p. 592). Although it had only 43 members, the VPK was the strongest grouping on the municipal council until 2002. Overall, there was much continuity with earlier forms of association. The VIM was regarded as innovative because it was not directly under the control of the MSZMP. Yet its activities built on those of club life within the framework of the Culture House since the mid-1970s. Particularly significant was the *Forrás és Új Tükör Klub*, named after journals published in Kecskemét and Budapest respectively, both of which featured reporting critical of conditions in late socialist society. No such clubs exist today. Critical local journalism, lively in the 1990s, has been similarly extinguished in the new century.

17 Today’s associations thus differ fundamentally from their presocialist antecedents, which were maintained entirely by their members. In 2019 the Kiskunhalas council distributed 2,000,000 Hungarian forints to 26 ‘civil organisations’ (including 150,000 to the Áron Szilády Society as a contribution to the costs of an excursion by coach to neighbouring Vojvodina). Only four applications were unsuccessful; one of these, submitted by the Pigeon-Fanciers Association, was redirected to a separate sport budget. Many of the allocations were to support a one-off event (these allocations were approved by the council on 25 April 2019: Kiskunhalas Város Képviselő-testülete, Kiskunhalas Város Önkormányzatának 2019. évi költségvetéséről szóló 7/2019. (II.22.) önkormányzati rendelete 2/b. mellékletében civil egyesületek egyéb működési célú kiadások jogcímen szereplő támogatásból a 2. melléklet szerint támogatja az abban szereplő civil szervezeteket, pályázókat).

18 Literally, ‘Hungarian Bourgeois/Civic’ Party. The name was changed again in 2003 to *Fidesz*—*Magyar Polgári Szövetség* (*Fidesz—*Hungarian Bourgeois/Civic Alliance). From 2006 to the present this party has been the dominant partner in a stable alliance with the Christian Democratic Party (*Kereszténydemokrata Néppárt*—KDNP).

19 See Bartha and Tóth, this issue.

20 On 26 July 2014 in a speech to ethnic Hungarians at Băile Tuşnad, Romania. See ‘Prime Minister Viktor Orbán’s Speech at the 25th Bálványos Summer Free University and Student Camp’, website of the Hungarian Government, 28 July 2014, available at: https://www.kormany.hu/en/the-prime-minister/the-prime-minister-s-speeches/prime-minister-viktor-orban-s-speech-at-the-25th-balvanyos-summer-free-university-and-student-camp, accessed 20 August 2020.

21 For a contemporaneous account by an American journalist, see Fleishman (2009).

22 Under the title ‘Mercedes-gyár épül Kecskeméten’, this item dominated the news coverage of the *Petőfi Népe* on 18 June 2008.

23 Fieldwork notes, 2018–2019.

24 It was reported to me locally in 2018–2019 that privatisation in 2004 had brought financial windfalls to a few Budapest managers, whose company collapsed within five years. This had drastic consequences for the operation of the hospital (both the treatment of patients and the payment of staff). By early 2010, socialist politicians in Halas were claiming that they too, and not only their equivalents in *Fidesz*, had played a full part bringing the hospital back under municipal control. See Fehér (2010).

25 Local elections are now organised every five years. Although diverse opposition parties united behind an independent in an effort to unseat the incumbent mayor, the latter received over two-thirds of the valid votes cast, compared with barely 50% in 2014, also in a three-way race. Turnout in October 2019 was 36%. *Fidesz*-endorsed candidates—including several respected personalities who were not members of the party—triumphed in every ward. In the European elections of 26 May 2019, turnout was just under 40% and the *Fidesz*–KDNP list obtained 57% of valid votes. Participation to elect members of parliament in Budapest is significantly higher (63.6% in the elections of 2018). See, ‘Polgármester-választás—Kiskunhalas’, available at: https://www.valasztas.hu/telepules-adatlap_onk2019?_onkeredmenyadatok_WAR_nvinvrportlet_formDate=32503680000000&p_p_id=onkeredmenyadatok_WAR_nvinvrportlet&p_p_lifecycle=1&p_p_state=normal&p_p_mode=view&p_p_col_id=column-2&p_p_col_pos=2&p_p_col_count=5&_onkeredmenyadatok_WAR_nvinvrportlet_tabId=tab2&p_r_p_1632770263_prpTelepulesKod=066&p_r_p_1632770263_prpVlId=294&p_r_p_1632770263_prpVltId=687&p_r_p_1632770263_prpMegyeKod=03&_onkeredmenyadatok_WAR_nvinvrportlet_prpVlId=294&_onkeredmenyadatok_WAR_nvinvrportlet_prpVltId=687&_onkeredmenyadatok_WAR_nvinvrportlet_prpMegyeKod=03&_onkeredmenyadatok_WAR_nvinvrportlet_prpTelepulesKod=066&_onkeredmenyadatok_WAR_nvinvrportlet_jelolesTipus=1&_onkeredmenyadatok_WAR_nvinvrportlet_evkSzam=, accessed 6 October 2021.

26 Halas has conformed to nationwide trends in this respect. The exposure of the deceitful promises Ferenc Gyurcsány made ahead of his victory in the 2006 national election was replicated in Halas by scandals surrounding a socialist member of parliament who was a native of the town.

27 See also, Szombati, this issue.

28 See also, Szombati, this issue.

29 *Provident* is a subsidiary of International Personal Finance, which prior to 2007 formed part of the Bradford-based Provident Financial Group. The British model of domestic loans mediated by locally-based agents who deal with debtors personally in their homes dates from the nineteenth century and has been successfully replicated throughout postsocialist Eastern Europe.

30 Author’s conversation, 18 September 2019.

31 Author’s conversation, 9 September 2019.

32 Author’s conversation, 9 September 2019.

33 The football team is sponsored by an entrepreneur whose business grew out of the privatisation of state farm ancillary activities. One player is an immigrant from West Africa who has married locally. While this sport has declined in recent years, the town’s handball team has enjoyed considerable success.

34 The major exception is the wine festival (*Szüreti Napok*) held during the second weekend of September, when national pop stars are joined by orchestras and dancers from twin towns in Poland and Germany. Many labour migrants return, combining family visits with larger parties. This festival grew out of celebrations originally organised by the Kiskunhalas State Farm. In the new century, to avoid inter-ethnic tensions, a separate festival is organised during the same weekend for the Roma minority. I have analysed this development as a prime example of postsocialist incivility (Hann 2020, pp. 475–77).

35 Author’s fieldwork, August and September 2015.

36 In discussion at the 1967 conference which led to the path-breaking publication *Populism. Its Meanings and National Characteristics* (Ionescu & Gellner 1969), Gellner considered the concept of populism too ‘nebulous’ for long-term reproduction. By contrast, Isaiah Berlin did not see anything ‘illogical or self-contradictory’ in populist power becoming entrenched. See, ‘To Define Populism’, The Isaiah Berlin Virtual Library, p. 21, available at: https://isaiah-berlin.wolfson.ox.ac.uk/sites/www3.berlin.wolf.ox.ac.uk/files/2020-12/Bib.111%28b-i%29%20-%20To%20Define%20Populism%20-%20transcript.pdf, accessed 18 September 2021.

37 I base this assertion primarily on fieldwork in summer 2016. The ‘migrant quota referendum’ of 2 October in that year resulted in over 98% voting ‘No’, as urged by the government, which consequently claimed an overwhelming victory. Opposition parties diagnosed a fiasco, since the low turnout rendered the outcome formally invalid. Several parties had urged a boycott or the spoiling of the voting form. Nevertheless, opinion polling throughout the year made it clear that a question posed in terms of national sovereignty could be answered in only one way by most citizens. For a thorough analysis of the campaign and results see, https://en.wikipedia.org/wiki/2016_Hungarian_migrant_quota_referendum, accessed 6 October 2021.

38 The theme of pseudo-civic organisations (*álcivil szervezetek*) already featured prominently in Viktor Orbán’s speech in July 2014 (see ‘Prime Minister Viktor Orbán’s Speech at the 25th Bálványos Summer Free University and Student Camp’, website of the Hungarian Government, 28 July 2014, available at: https://www.kormany.hu/en/the-prime-minister/the-prime-minister-s-speeches/prime-minister-viktor-orban-s-speech-at-the-25th-balvanyos-summer-free-university-and-student-camp, accessed 20 August 2020; see also Hann 2020, pp. 477–78).

39 The classical study of Western orientalising discourses about Eastern Europe is Wolff (1994). Stereotypes have been revived in postsocialist decades, not only in the West but also by liberals in the East seeking explanations for the democratic shortcomings of their compatriots; for a notable early example, see Sztompka (1993); a more systematic account of the ‘east-west slope’ is provided by Melegh (2006). For an interesting study of how Viktor Orbán himself has practised innovative forms of ‘self-Orientalism’, always with a view to his audiences in the West as well as domestically, see Rac (2014).

40 This legislation was part of a package known as ‘Stop Soros’ that featured prominently in the election campaign of Spring 2018 before being approved by the newly elected parliament on 20 June. See, ‘2018. évi VI. Törvény: egyes törvényeknek a jogellenes bevándorlás elleni intézkedésekkel kapcsolatos módosításáról’, available at: http://www.kozlonyok.hu/nkonline/MKPDF/hiteles/MK18097.pdf, accessed 6 October 2021. For a commentary see, ‘On World Refugee Day, Hungary Makes it Illegal to Help Refugees and Migrants’, Open Society Foundations, 18 June 2018, available at: https://www.opensocietyfoundations.org/newsroom/world-refugee-day-hungary-makes-it-illegal-help-refugees-and-migrants/, accessed 20 September 2021.

41 Government attitudes to Merkel have been more restrained in the wake of the clashes provoked by the refugee flows in summer 2015. Orbán himself has been careful to avoid criticism in public, since he depends on German backing to enable the continued flow of EU transfers (liberals both in the West and within Hungary criticise Merkel for putting up with this manipulation). But widespread negative opinions about the long-serving chancellor were accurately reflected in a 2019 interview with Mária Schmidt, an Orbán confidante, conducted by Collin McMahon; see: ‘Victor Orban Adviser Maria Schmidt: “Orban and Trump are like Twins”’, *Gateway Pundit*, 11 June 2019, available at: https://www.thegatewaypundit.com/2019/06/victor-orban-adviser-maria-schmidt-orban-and-trump-are-like-twins/, accessed 6 October 2021. See also the reporting of Bakos (2017) ahead of the German federal elections, in which Merkel is depicted as a ‘marionette’. The campaign against Soros was revived in late 2020 (Walker 2020).
